# Efficacy of mizoribine in renal transplant recipients on calcineurin inhibitor-based immunosuppression: a network meta-analysis

**DOI:** 10.3389/fmed.2026.1714190

**Published:** 2026-02-18

**Authors:** Bohan Luo, Bo Yang, Changtao Zhong, Han Luo

**Affiliations:** 1 School of Medicine, University of Electronic Science and Technology of China, Chengdu, China; 2 Xindu District Hospital of Traditional Chinese Medicine, Chengdu, China; 3 Department of Hepatobiliary Surgery, Zigong Fourth People's Hospital, Zigong, China

**Keywords:** calcineurin inhibitor, efficacy, immunosuppression, mizoribine, Network meta-analysis, renal transplantation, safety

## Abstract

**Objective:**

To systematically evaluate the efficacy of mizoribine (MZR) in renal transplant recipients on a calcineurin inhibitor (CNI)-based maintenance regimen and to compare it with other immunosuppressants [mycophenolic acid (MPA), mycophenolate mofetil (MMF), cyclophosphamide (CTX)] utilizing network meta-analysis (NMA).

**Materials and methods:**

Randomized controlled trials (RCTs) of MZR and other immunosuppressants in renal transplant recipients were retrieved from databases including PubMed, Web of Science, and Science Direct. Study quality was assessed. NMA was performed utilizing *RevMan 5.3* and *Stata 18.0*, generating surface under the cumulative ranking curve (SUCRA) values to compare treatments based on efficacy, safety, and patient survival.

**Results:**

A total of 11 studies were included. Within CNI-based maintenance regimens, MZR showed no significant differences compared to MPA, MMF, or CTX in terms of patient survival (3-year) or graft survival (*P* > 0.05). Surface under the SUCRA analysis indicated that MPA ranked highest for both patient survival (69.8%) and graft survival (69.4%), followed by MZR (61.3 and 58.4%, respectively). Regarding renal function, as indicated by serum creatinine levels, no significant difference was observed between MZR and MMF. In the SUCRA ranking for this outcome, CTX was optimal (90.4%), with MZR ranking moderate (55.3%). For the incidence of acute rejection, no significant differences were found among the agents, although SUCRA values suggested MMF might be most favorable (88.2%), while MZR ranked lowest (21.7%). In terms of safety, the incidence of gastrointestinal adverse events (AEs) was significantly lower with MZR than with the other drugs (SUCRA: 90.2%). However, MZR was associated with a higher risk of BK virus viremia (SUCRA: 16.2%). The incidence of leukopenia with MZR was comparable to that with MMF, though MZR had a lower SUCRA ranking (54.2%) for this outcome.

**Conclusion:**

In CNI-based maintenance therapy for renal transplant recipients, MZR is equivalent to MPA and MMF in ensuring long-term patient and graft survival. It exhibits a distinct safety profile: significantly superior gastrointestinal tolerability and a lower risk of leukopenia, albeit with a potentially higher risk of BK viremia. In terms of renal function, MZR demonstrates an intermediate effect, superior to MMF but inferior to CTX. Clinical selection should involve weighing the risks of infection (particularly BK virus) against gastrointestinal tolerability based on individual patient characteristics. MZR represents an effective and well-tolerated important alternative to traditional MMF/MPA.

## 1 Introduction

Renal transplantation is an effective treatment for patients with end-stage renal disease, significantly improving survival rates (1). Long-term success after transplantation highly depends on effective immunosuppressive regimens to prevent graft rejection. calcineurin inhibitor (CNI)-based immunosuppressive protocols, utilizing agents such as tacrolimus or cyclosporine, form the cornerstone of current maintenance therapy following renal transplantation (2). However, long-term use of CNIs can cause numerous adverse effects, most notably nephrotoxicity, neurotoxicity, and metabolic disorders, which may compromise graft function and impact long-term patient survival (3, 4).

To reduce CNI exposure and its associated toxicity while maintaining immunosuppressive efficacy, combination therapy strategies are commonly employed in clinical practice, which involve adding other auxiliary immunosuppressants to a CNI backbone. Mycophenolate mofetil (MMF) is currently the most widely used auxiliary agent, but its adverse effects, such as gastrointestinal toxicity and myelosuppression, limit its use in some patients (5). Consequently, identifying alternative or adjunctive immunosuppressants with high efficacy and superior safety profiles is of significant clinical importance. Mizoribine (MZR), a purine analog, exerts immunosuppressive effects by inhibiting lymphocyte proliferation. It has been used for anti-rejection therapy after renal transplantation in Asian countries such as Japan and China (6, 7). Compared to MMF, some studies suggested that MZR may offer a distinct safety profile, particularly better gastrointestinal tolerability (8). Moudgil et al. (9) report that MMF is an effective immunosuppressant for preventing acute rejection in renal transplant recipients. Quiroz et al. (10) indicated that maintaining mycophenolic acid (MPA) levels within the therapeutic range with MMF was well-tolerated, without notable adverse events (AEs), and resulted in stable renal function throughout the follow-up period. Yatim et al. (11) found that preemptive reduction of MMF dose could be an effective strategy to prevent infections during long-term MMF administration. Nevertheless, conclusions from existing individual studies on efficacy of MZR in CNI-based regimens are inconsistent, and there is a lack of systematic reviews directly comparing it with other treatment options (such as MMF, sirolimus, or placebo).

To better evaluate the clinical value of MZR in renal transplant recipients on a CNI-based regimen, this study employed a network meta-analysis (NMA) approach. NMA allows for integration of various evidences to quantitatively rank multiple interventions. This study comprehensively compared the relative efficacy of MZR with other adjunctive treatments regarding reducing acute rejection rates, preserving graft renal function, and the risk of AEs, thereby providing a foundation for clinical decision-making and supporting the development of individualized treatment strategies.

## 2 Materials and methodologies

### 2.1 Criteria

This study was conducted in accordance with the PRISMA guidelines (12). The eligibility criteria were established based on the PICOS framework as follows:

Inclusion criteria: (i) study type: randomized controlled trials (RCTs), irrespective of blinding status (non-blinded studies were included given that open-label designs are common and blinding is often challenging to implement in trials of immunosuppressive agents, and therefore blinding did not serve as an exclusion criterion); (ii) participants: adult renal transplant recipients receiving a CNI (e.g., tacrolimus or cyclosporine)-based immunosuppressive regimen; (iii) interventions: the experimental group received a regimen containing MZR, while the control group received other adjunctive immunosuppressants [MPA, MMF, and cyclophosphamide (CTX)], with an intervention and follow-up duration ≥3 months; (iv) outcome measures: primary outcomes included the incidence of acute rejection and estimated glomerular filtration rate (eGFR); secondary outcomes included patient survival, graft survival, overall AEs (AEs), infections (e.g., cytomegalovirus infection), leukopenia, and gastrointestinal AEs.

Exclusion criteria: (i) studies involving multi-organ transplant or re-transplant recipients; (ii) non-Chinese or non-English publications; (iii) duplicate publications, conference abstracts, commentaries, case reports, reviews, and studies with unavailable or incomplete data; (iv) studies with unclear interventions or those combining other experimental drugs.

### 2.2 Search strategy

A systematic search was conducted in PubMed, Web of Science, and Science Direct, covering the period from their inception to May 2025. The search strategy combined subject headings and free-text terms utilizing Boolean operators (“AND,” “OR”). The search strategy for PubMed is illustrated as follows:

((((“Kidney Transplantation”[Mesh]) OR (Renal Transplant^*^[Title/Abstract])) AND ((“calcineurin inhibitors”[Mesh]) OR (Tacrolimus[Title/Abstract]) OR (Cyclosporine[Title/Abstract]))) AND ((“Mizoribine”[Mesh]) OR (Mizoribine[Title/Abstract]) OR (Bredinin[Title/Abstract]))) AND ((“Randomized Controlled Trial”[Publication Type]) OR (Randomized[Title/Abstract]) OR (controlled clinical trial[Title/Abstract])). Search strategies for other databases were adapted according to their respective controlled vocabularies.

### 2.3 Screening and data extraction

Two researchers independently screened the retrieved literature. Duplicate records were removed utilizing *EndNote* (Clarivate Analytics, USA). The initial screening was performed by reviewing titles and abstracts to exclude obviously irrelevant studies. Full-text articles were obtained and reviewed for final inclusion. Any discrepancies were resolved through discussion or by consulting a third researcher.

### 2.4 Quality evaluation

The methodological quality of the included RCTs was assessed using the Cochrane Risk of Bias tool (RoB 2.0) (13), which evaluates the following five domains: (i) bias arising from the randomization process; (ii) bias due to deviations from intended interventions; (iii) bias due to missing outcome data; (iv) bias in measurement of the outcome; and (v) bias in selection of the reported result. The risk of bias for each domain was judged as “low risk,” “high risk,” or “some concerns.”

### 2.5 Extraction of literature materials

Data were independently extracted from the finally included studies by two investigators utilizing a pre-designed standardized data extraction form. The extracted information included: (i) basic study characteristics: first author, publication year, journal, country/region where the study was conducted, study design (e.g., single-center/multi-center), and follow-up duration. (ii) Participant characteristics: sample size of each group, baseline data such as mean age, sex ratio, composition of primary diseases, and time post-transplantation. (iii) Interventions: specific details of intervention protocols in experimental and control groups (e.g., MZR dosage, frequency of administration, type of CNI and target blood concentration range, concomitant use of steroids). (iv) Outcome data: data for primary outcomes (e.g., number of acute rejection events, mean and standard deviation of eGFR) and secondary outcomes (e.g., number of patient survival and graft survival events, incidence of various AEs). After extraction, a third researcher verified the accuracy of the data. In cases of missing or ambiguously reported data, the corresponding authors of publications were contacted via email to obtain information. If unavailable, the situation was documented and addressed in the analysis.

### 2.6 Statistical methodologies

Traditional pairwise meta-analysis and generation of risk of bias assessment graphs were performed utilizing *RevMan 5.3* (The Cochrane Collaboration, Nordic Cochrane Center). NMA was conducted utilizing the network suite of commands in *Stata MP 18.0* (StataCorp LLC, USA). For dichotomous outcomes (e.g., incidence of acute rejection), the odds ratio (OR) served as effect measure; for continuous outcomes (e.g., eGFR), the mean difference (MD) was used, both presented with their 95% confidence intervals (CI). A network geometry plot was drawn to visually represent the direct comparison relationships between various interventions, where node size corresponds to sample size and thickness of connecting lines represents the number of studies available for each direct comparison. If closed loops existed in the network, the node-splitting method or the inconsistency factor approach was employed to assess consistency between direct and indirect evidence. If *P* > 0.05, a consistency model was applied; if inconsistency was detected, an inconsistency model was used, accompanied by sensitivity analyses to explore sources of heterogeneity. The surface under the cumulative ranking curve (SUCRA) (14) was calculated to rank the interventions for each outcome in terms of efficacy or safety. A higher SUCRA value (expressed as a percentage) indicates a higher ranking and a greater probability of being the best intervention. Eventually, comparison-adjusted funnel plots were used to assess potential small-study effects or publication bias.

## 3 Results

### 3.1 The retrieval process of literature

**Figure 1:**
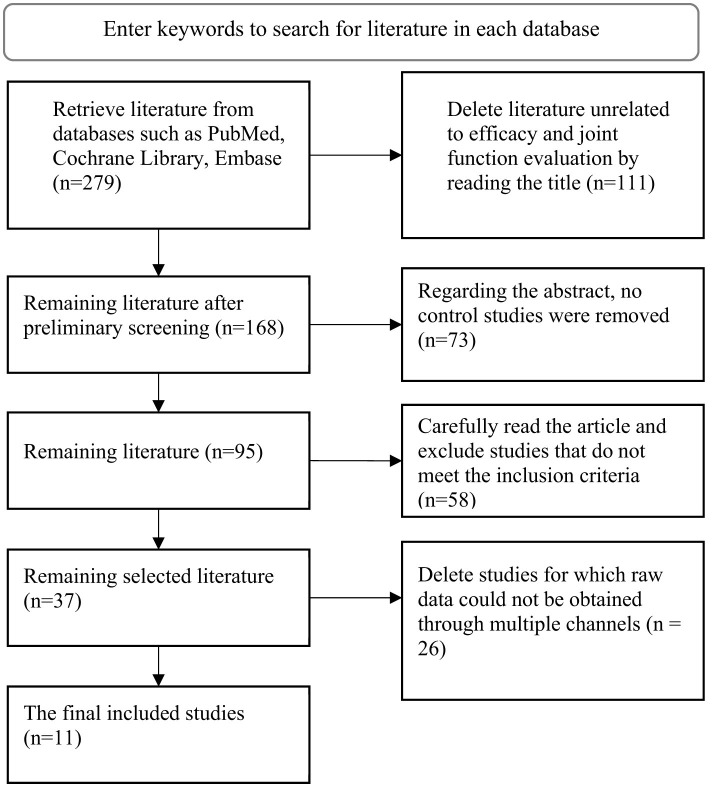
Basic process of literature search.

A systematic literature search was performed using predefined keyword combinations across major online databases, which initially yielded 279 relevant articles. The literature screening process consisted of four sequential stages. First, focusing on the relevance between the article titles and the research evaluation indicators, 111 studies unrelated to the topic were excluded, resulting in 168 articles retained preliminarily. Next, during the “detailed abstract review,” the integrity of the study design was emphasized, and 73 articles lacking control groups or not involving analysis of influencing factors were excluded, leaving 95 articles to proceed to the next stage. Subsequently, a “full-text assessment” was conducted based on pre-established inclusion criteria (such as study type, subject population, outcome measures, etc.), and full texts of 95 articles were reviewed one by one. This led to the exclusion of 58 articles, with 37 articles remaining. In “original data verification” phase, the 37 included articles were thoroughly examined. An additional 26 articles for which key original data (such as effect sizes, sample sizes, statistical indicators, etc.) could not be obtained were excluded, ultimately resulting in 11 articles being included. The complete process of literature retrieval and screening for this study is detailed in Figure 1.

### 3.2 Basic information

After multi-stage screening, 11 studies were ultimately included for quantitative synthesis, nine of which provided extractable subgroup data (such as BK viremia, living-donor transplantation, ABO incompatibility, etc.). The total sample size comprised 497 patients. Interventions included: MZR, tacrolimus, MMF, and MPA (Table 1).

**Table 1:** Basic information of literature.

**No**.	**Author**	**Year**	**MZR group/control group**	**Treatment method**	**Outcome measure**
1	Shi (15)	2019	22/20	MZR vs. MMF	a, b, c, d, h
2	Peng (16)	2020	36/37	MZR vs. MMF	a, b, c, e, f
3	Wang (17)	2023	30/30	MZR vs. MPA	a, d
4	Li (18)	2025	22/28	MMF vs. MPA	f
5	Zheng (19)	2023	155/99	MPA vs. MZR	e, g, f, h
6	Yoshimura (20)	2014	12/12	MZR vs. MMF	a, b, d, e, g, h
7	Dong (21)	2025	123/120	MZR vs. CTX	e
8	Huang (22)	2025	46/46	MMF vs. MZR	a, c, f
9	Shi (23)	2017	22/20	MZR vs. MMF	a, c, d, g, h
10	Ishida (24)	2016	41/42	MZR vs. MMF	a, b, c, e, g, h
11	Harada (25)	2017	22/15	MZR vs. MPA	a, b, c, d, e, h

Outcome indicators: a: patient survival rate; b: graft survival rate; c: acute rejection; d: renal function; e: overall infection; f: BK viremia; g: leukopenia; h: gastrointestinal AEs.

### 3.3 Quality evaluation

**Figure 2:**
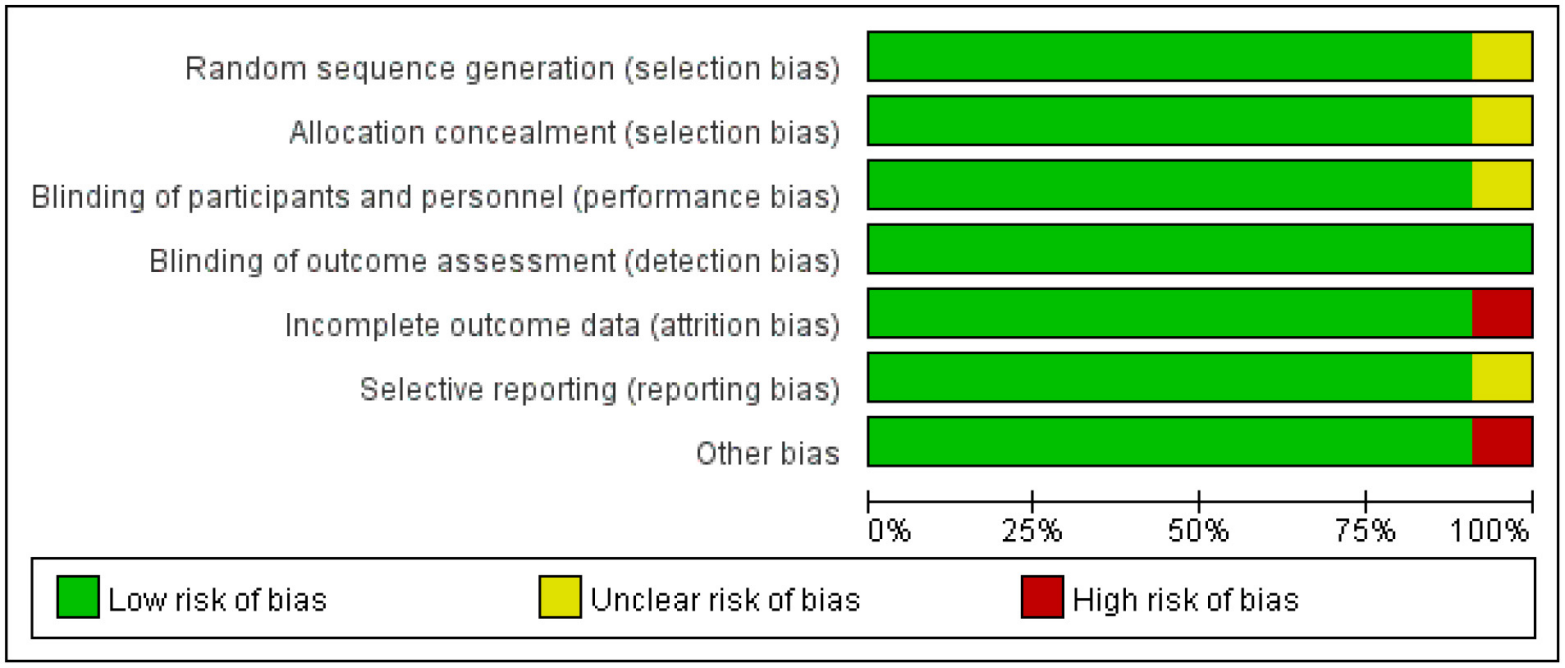
Bar chart for literature bias risk assessment (assessment based on Cochrane RoB 2.0; figure annotations use conventional terminology).

**Figure 3:**
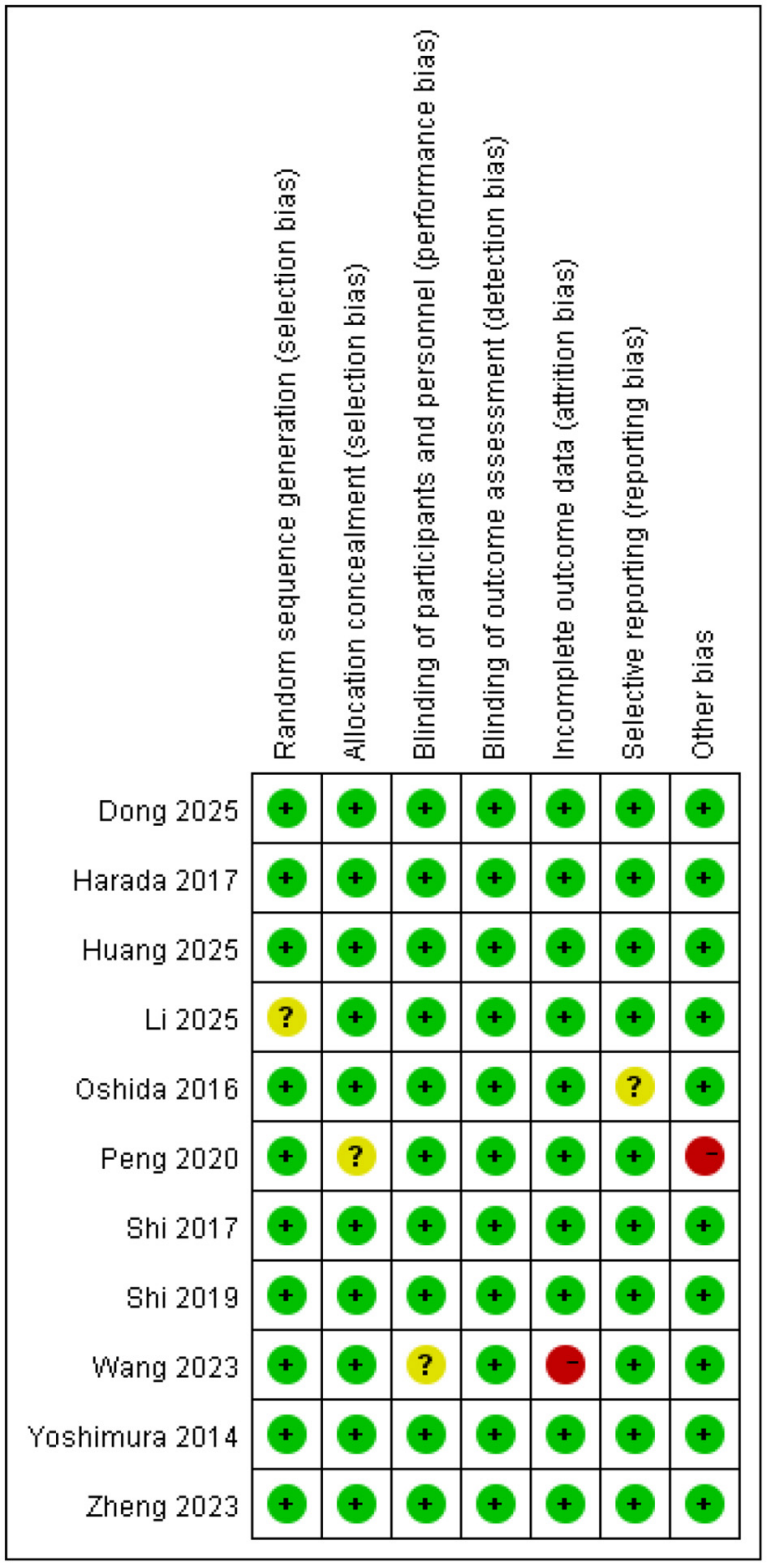
Summary of literature bias risk assessment (assessment based on RoB 2.0; conventional terminology used).

Based on the Cochrane RoB 2.0 assessment tool, the risk of bias for the included RCTs was evaluated, and the results are presented graphically in Figures 2, 3. To facilitate comparison with conventional assessment dimensions, the figures retain classic terminology (e.g., selection bias, detection bias) for annotation. The correspondence between these terms and the actual RoB 2.0 assessment domains is as follows: “Random sequence generation (selection bias)” corresponds to bias arising from the randomization process; “Allocation concealment (selection bias)” also corresponds to bias arising from the randomization process; “Blinding of outcome assessment (detection bias)” corresponds to bias in the measurement of the outcome; “Incomplete outcome data (attrition bias)” corresponds to bias due to missing outcome data; and “Selective reporting (reporting bias)” corresponds to bias in the selection of the reported result. The assessment results showed that all studies were judged to have a “low risk” of bias concerning incomplete outcome data and selective reporting. For random sequence generation, one study was rated as “unclear.” Regarding allocation concealment, one study was rated “low risk” and another “high risk.” For the blinding of outcome assessment, one study was judged as “unclear.” Concerning other biases, two studies were rated “unclear.”

### 3.4 NMA of surgical efficacy outcomes

#### 3.4.1 Survival rate

**Figure 4:**
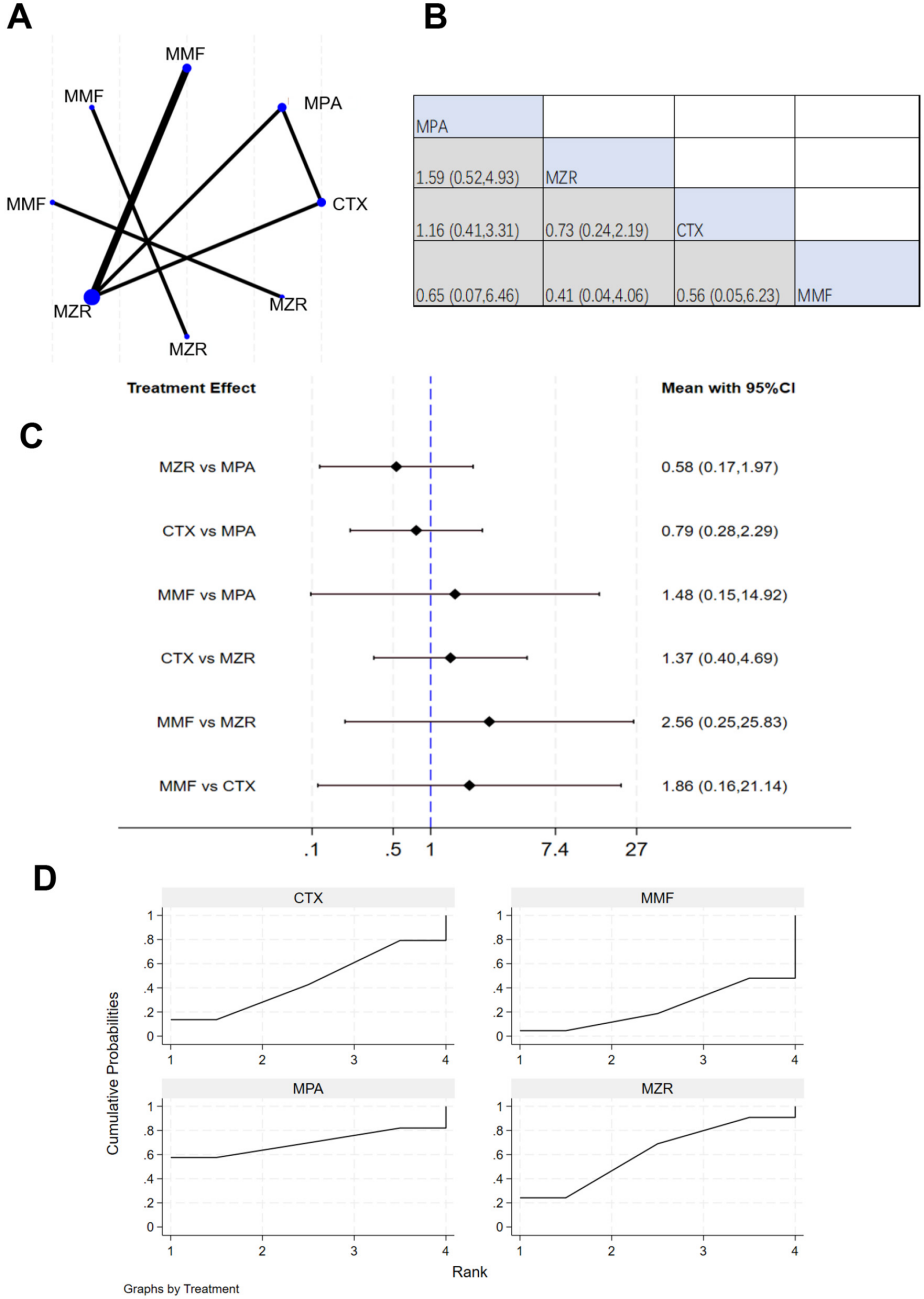
NMA results of patient survival. **(A)** Network evidence relationship diagram for survival; **(B)** league table for survival; **(C)** NMA results for survival; **(D)** SUCRA plot for survival. In **(A)** the size of the nodes is proportional to the sample size, and the thickness of the connecting lines corresponds to the number of available direct comparison studies. Direct comparative evidence exists between MZR and MMF, whereas the comparison between MZR and MPA is based solely on indirect evidence, as no direct comparison studies were identified.

Eleven studies reported 3-year survival data. The network evidence relationship (NER) diagram is shown in Figure 4A. Figure 4B indicates that negligible differences existed in patient survival rates between any pairwise comparisons among the four immunosuppressants (MPA, MZR, CTX, and MMF). The results in Figure 4C show that the comparison of MZR vs. MPA had a 95% CI of 0.17–1.97, suggesting slight difference in survival impact between MZR and MPA (*P* > 0.05). The comparison of “MMF vs. MZR” yielded a 95% CI of 0.25–25.83, indicating a potential difference in survival impact between MMF and MZR (*P* < 0.05). The SUCRA ranking for survival rates in Figure 4D was: MPA (69.8%) > MZR (61.3%) > CTX (45.2%) > MMF (23.7%).

#### 3.4.2 Transplant survival rate

**Figure 5:**
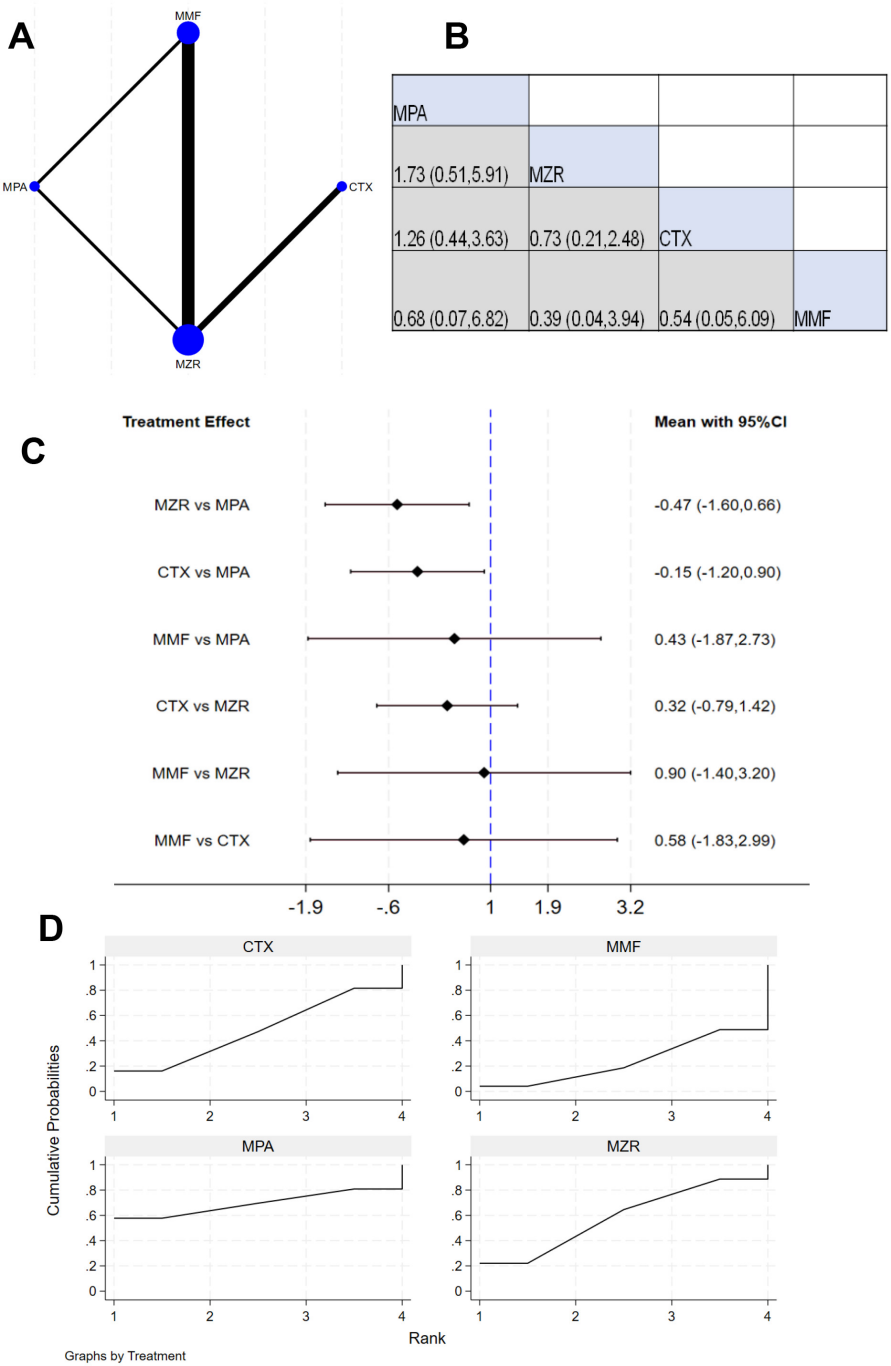
NMA results of transplant survival. **(A)** NER diagram of transplant survival; **(B)** league table of transplant survival; **(C)** NMA results of transplant survival in kidney transplant patients; **(D)** SUCRA plot of transplant survival.

Six studies reported data on the survival rate of kidney transplant patients. The NER diagram for kidney transplant patient survival is shown in Figure 5A. Figure 5B indicates that the cell at the intersection of MPA (row) and MZR (column) displays an effect size of 1.73 (0.51, 5.91), meaning that relative MZR, MPA has a relative effect of 1.73 (95% CI: 0.51–5.91) on survival-related outcomes. The results in Figure 5C show that the mean effect for MZR vs. MPA was −0.47 (−1.80, 0.66), and for MMF vs. MPA was 0.43 (−1.87, 2.73). This confirms that no drug demonstrated a marked advantage in improving transplant survival. The SUCRA ranking for transplant survival in Figure 5D was: MPA (69.4%) > MZR (58.4%) > CTX (48.3%) > MMF (23.8%).

#### 3.4.3 Acute rejection rate

**Figure 6:**
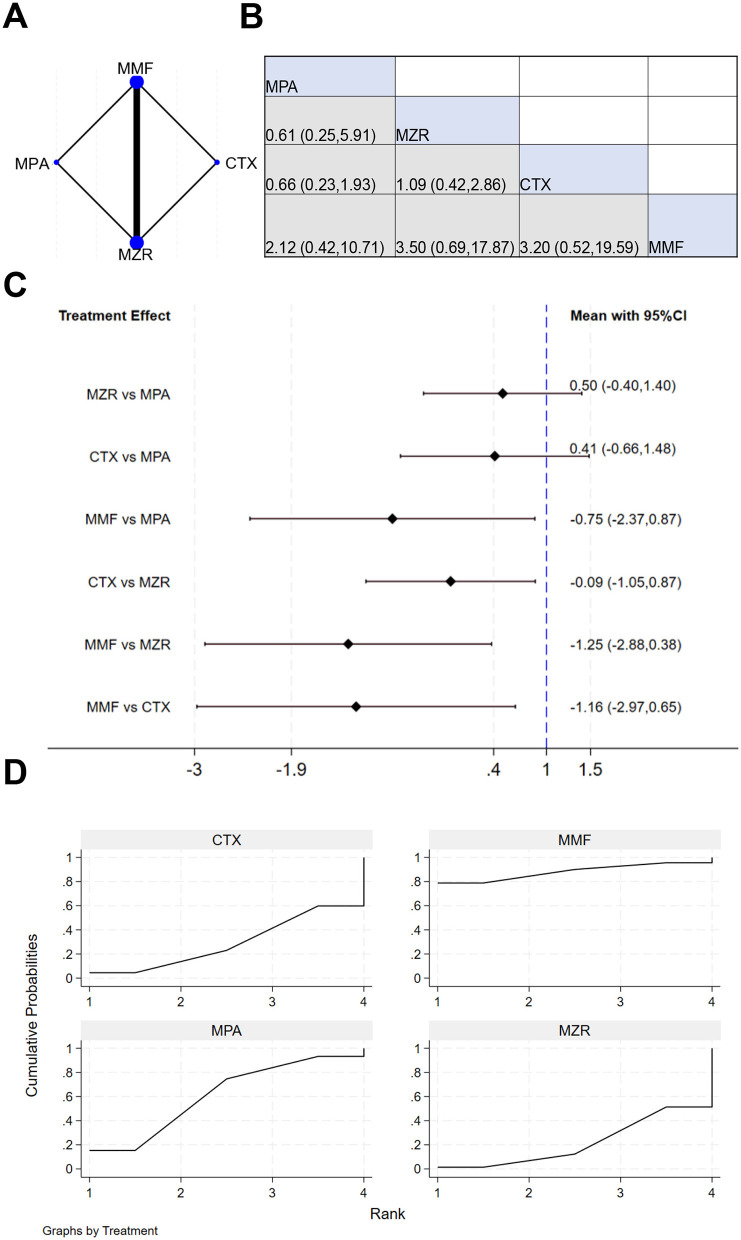
NMA results of acute rejection in patients. **(A)** NER plot of acute rejection rate; **(B)** league table of incidence of acute rejection reactions; **(C)** NMA results on the incidence of acute rejection reactions; **(D)** SUCRA plot of incidence of acute rejection reaction.

A total of six studies reported data on the incidence of acute rejection in kidney transplant patients. NER diagram for acute rejection incidence is shown in Figure 6A, indicating direct comparison connections between MZR and MMF, MPA and MMF, and MPA and CTX, suggesting that direct clinical study data support comparisons between these drugs. Figure 6B shows that the 95% CI for “MPA vs. MZR” is 0.61 (0.25, 5.91), indicating negligible difference between MPA and MZR in the study outcomes (*P* > 0.05). The results in Figure 6C demonstrate that the 95% CI for “MZR vs. MPA” is −0.40 to 1.40, suggesting no difference in the impact of MZR and MPA on the outcomes (*P* > 0.05). The SUCRA ranking for the incidence of acute rejection in Figure 6D is: MMF (88.2%) > MPA (61.0%) > CTX (29.1%) > MZR (21.7%).

#### 3.4.4 Infection rate

**Figure 7:**
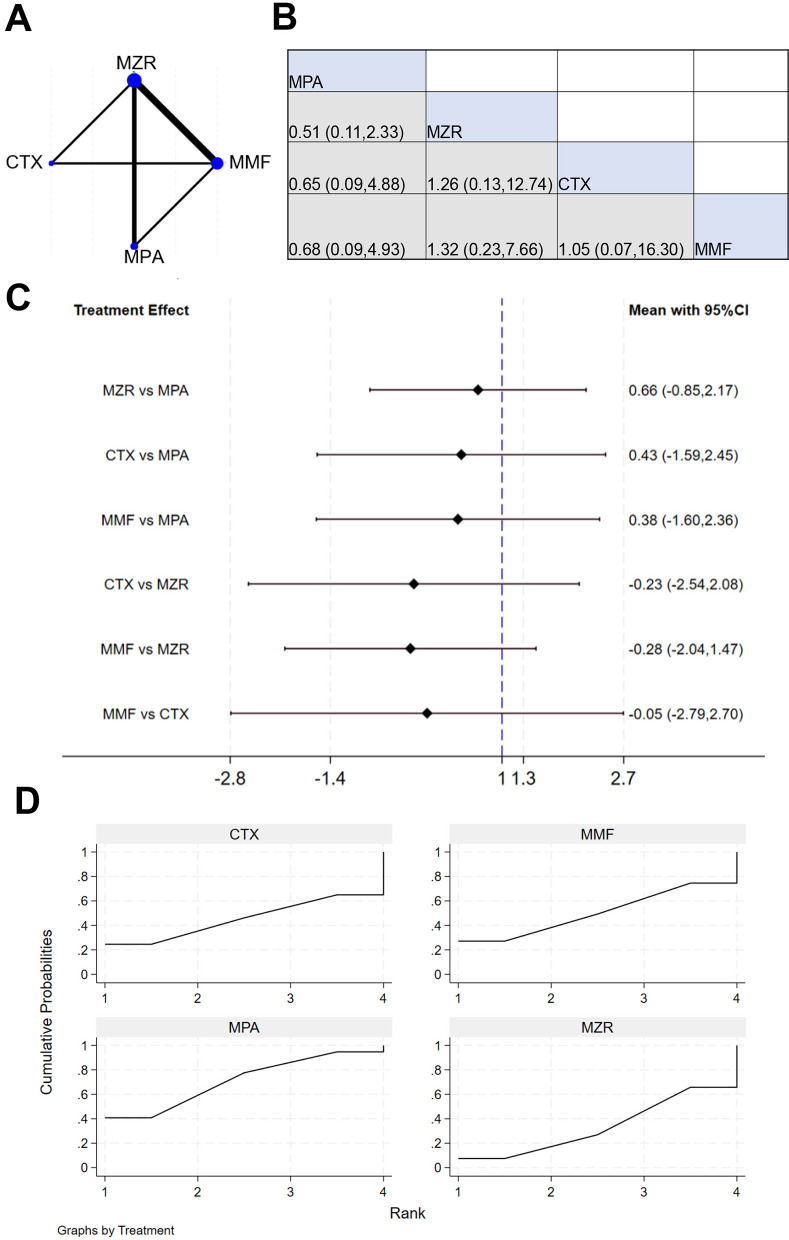
NMA results of patient infection rate. **(A)** infection rate chart; **(B)** league table of infection rates; **(C)** NMA results of infection rate; **(D)** SUCRA chart of infection rate.

A total of six studies reported data on the infection rate in kidney transplant patients. The NER diagram for infections in kidney transplant patients is shown in Figure 7A. Figure 7B displays the effect size and 95% CI for MMF vs. MZR [1.32 (0.23, 7.33)], indicating insignificant difference in infection rates between the two (*P* > 0.05). The results in Figure 7C show the effect size and 95% CI for MMF vs. MZR [−0.28 (−2.04, 1.47)], again demonstrating no significant difference in infection rates (*P* > 0.05). The SUCRA ranking for infection rates in Figure 7D is: MPA (71.0%) > MMF (50.3%) > CTX (45.2%) > MZR (33.4%).

#### 3.4.5 Incidence of BK viremia

**Figure 8:**
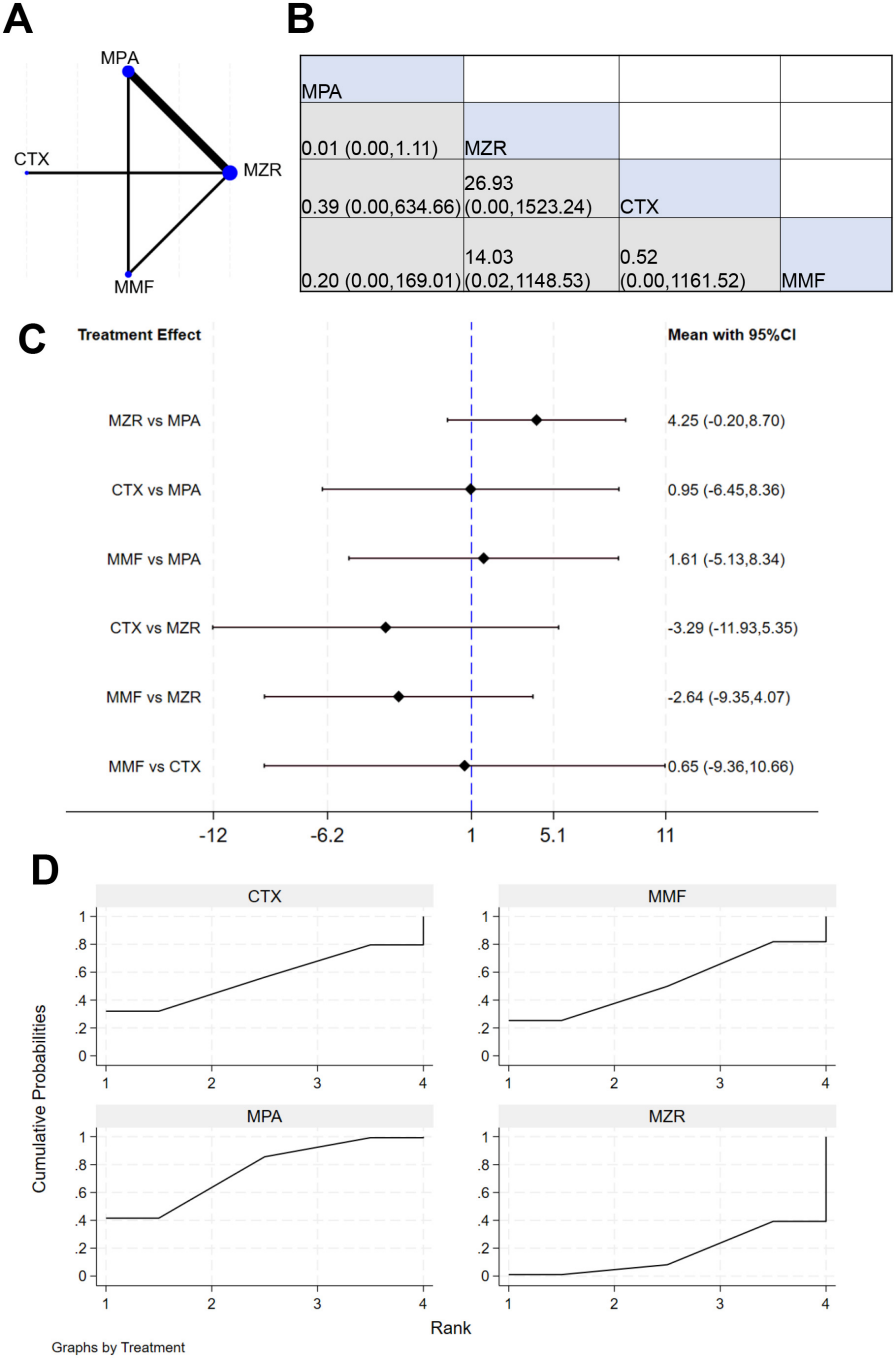
NMA results of BK viremia incidence. **(A)** NER diagram of BK viremia incidence; **(B)** league table of BK viremia incidence; **(C)** NMA results of BK viremia incidence; **(D)** SUCRA plot of BK viremia incidence.

A total of four studies reported data on the incidence of BK viremia in kidney transplant patients. The NER diagram for BK viremia incidence is shown in Figure 8A. The results in Figures 8B, C indicate negligible difference in BK viremia incidence between MZR and MMF (*P* > 0.05). The SUCRA ranking for BK viremia incidence in Figure 8D is: MPA (75.5%) > CTX (56.0%) > MMF (52.3%) > MZR (16.2%).

#### 3.4.6 Incidence of leukopenia

**Figure 9:**
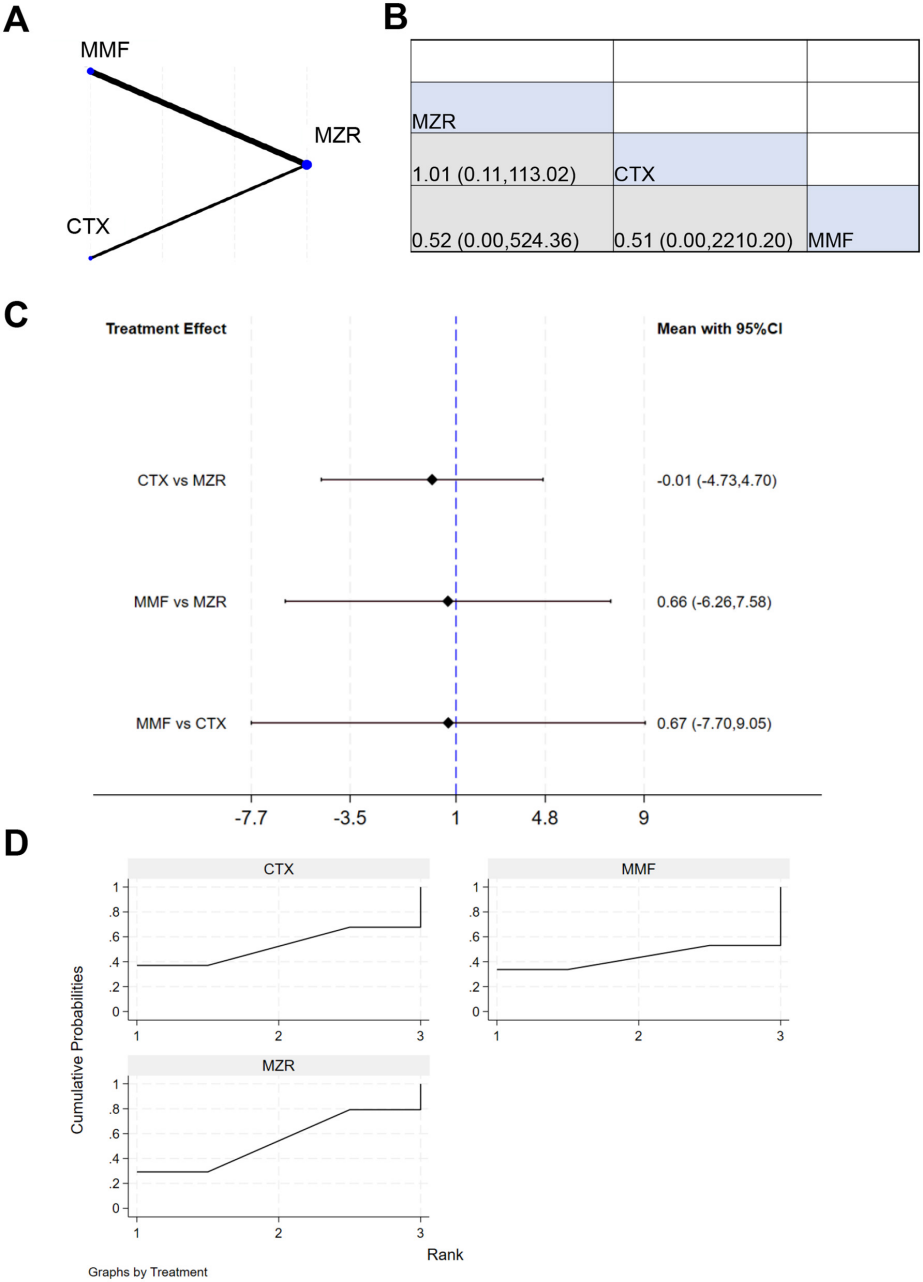
NMA results of incidence of leukopenia in patients. **(A)** NER plot of the impact on the incidence of leukopenia; **(B)** league table of incidence of leukopenia; **(C)** NMA results on the incidence of leukopenia; **(D)** SUCRA graph of the impact of leukopenia incidence rate.

Nine studies reported data on leukopenia incidence in kidney transplant patients. The NER diagram for leukopenia is shown in Figure 9A. The results in Figures 9B, C indicate slight difference in leukopenia incidence between MZR and MMF (*P* > 0.05). SUCRA ranking for the incidence of leukopenia in Figure 9D is: MZR (54.2%) > CTX (52.4%) > MMF (43.4%).

#### 3.4.7 Incidence of gastrointestinal dysfunction events

**Figure 10:**
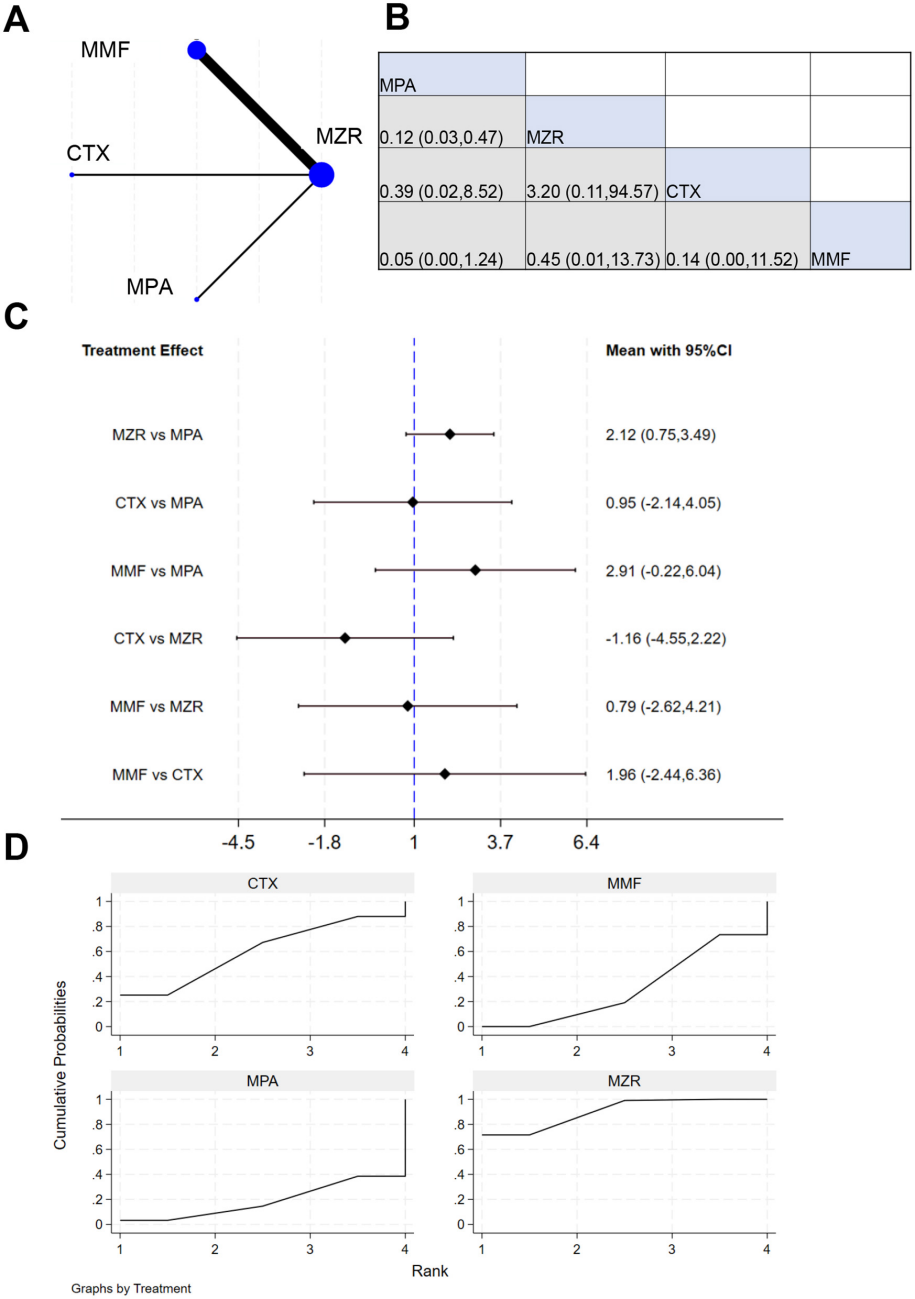
NMA results of incidence of gastrointestinal AEs in patients. **(A)** NER plot of incidence of gastrointestinal AEs; **(B)** league table of incidence of gastrointestinal dysfunction events; **(C)** NMA results on the incidence of gastrointestinal functional AEs; **(D)** SUCRA chart of incidence of gastrointestinal functional AEs.

Seven studies reported data on incidence of gastrointestinal AEs in kidney transplant patients. The NER diagram for gastrointestinal AEs is shown in Figure 10A. The results in Figures 10B, C indicate insignificant difference in incidence of gastrointestinal AEs between MZR and MMF (*P* > 0.05). SUCRA ranking for the incidence of gastrointestinal AEs in Figure 10D is: MZR (90.2%) > CTX (60.1%) > MMF (30.9%) > MPA (18.8%).

#### 3.4.8 Serum creatinine

**Figure 11:**
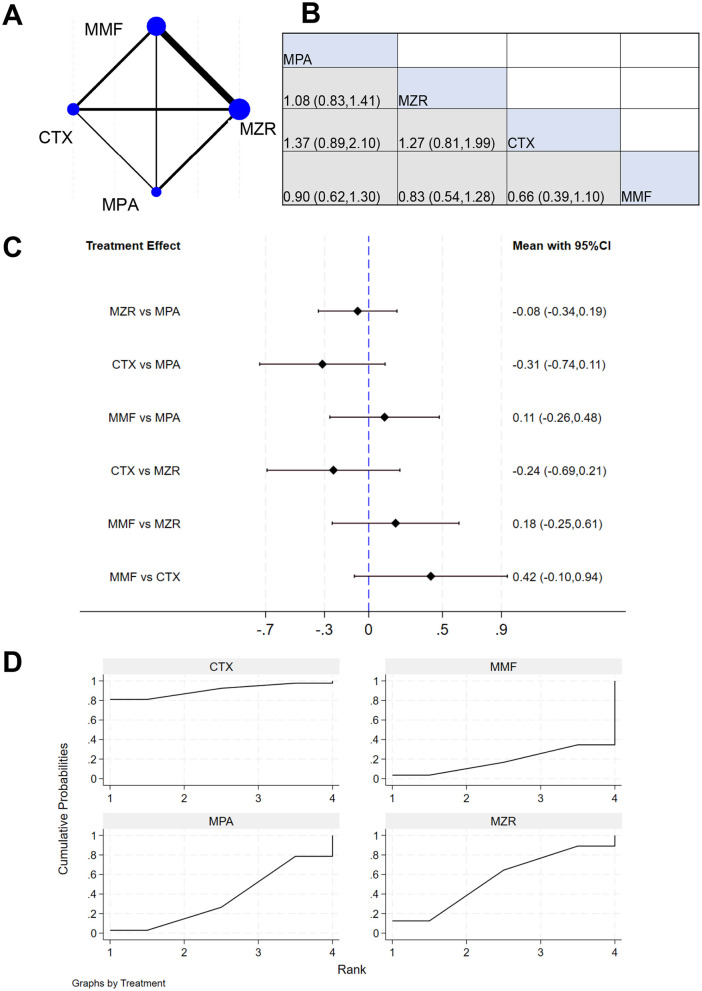
NMA results of patient serum creatinine. **(A)** NER plot of serum creatinine; **(B)** league table of serum creatinine; **(C)** NMA results of serum creatinine; **(D)** SUCRA plot of serum creatinine.

A total of six studies reported serum creatinine data in kidney transplant patients. The NER diagram for serum creatinine data is shown in Figure 11A, with the network structure providing the basis for subsequent analysis. The results in Figures 11B, C show that the effect size for MPA vs. MZR was 1.08, with a 95% CI of (0.83, 1.41); the effect size for CTX vs. MZR was 1.37, with a 95% CI of (0.89, 2.10). Negligible difference was observed between MZR and MMF in serum creatinine data (*P* > 0.05). The treatment effect for MMF vs. MZR was 0.18, with a 95% CI of (−0.25, 0.61), implying negligible difference (*P* > 0.05). The SUCRA ranking for serum creatinine in Figure 11D was: CTX (90.4%) > MZR (55.3%) > MPA (36.0%) > MMF (18.3%).

### 3.5 Heterogeneity and publication bias

The node-splitting method indicated inconsiderable inconsistency (*P* = 0.38). The funnel plot exhibited good symmetry, and Egger's test revealed negligible publication bias (*P* = 0.074).

## 4 Discussion

Our work conducted a Bayesian NMA to comprehensively compare efficacy and safety of MZR with other mainstream immunosuppressants (MPA, MMF, and CTX) in CNI-based maintenance regimens for kidney transplant recipients. The main findings revealed that MZR demonstrated comparable efficacy to other drugs in ensuring long-term survival of transplant recipients and grafts, while its safety profile exhibited a dual nature of unique advantages and risks, providing important evidence-based support for individualized clinical decision-making. No marked differences were observed among MZR, MPA, MMF, and CTX regarding patient survival and graft survival rates. These results indicate that, within CNI-based regimens, MZR as an antimetabolite exhibits immunosuppressive potency and effects equivalent to MMF/MPA in achieving the fundamental goal of preventing graft loss and patient mortality. In the SUCRA rankings, MPA slightly outperformed MZR in two outcome measures, though without statistical support. This suggests a potential trend toward MPA being the most effective option, though higher-quality studies are required to verify reliability of this trend, consistent with some published studies (26, 27), reinforcing the role of MZR as a valid alternative immunosuppressant. Regarding the incidence of acute rejection, while direct comparisons showed slight differences, the SUCRA ranking (MMF > MPA > CTX > MZR) warrants further investigation. The lowest ranking of MZR may indicate relatively weaker efficacy in preventing acute rejection, though this must be interpreted with caution. Given the lack of statistical significance in all comparisons and the absence of differences in survival outcomes, the observed ranking disparities are more likely attributable to variations in patient characteristics, dosing regimens, or concomitant medications across the included studies, rather than absolute differences in drug efficacy. In clinical practice, factors such as MZR dosing (e.g., 25–50 mg/kg/day) and therapeutic drug monitoring may critically influence outcomes. A limitation of this analysis is the inability to perform subgroup analyses based on dosage.

The most clinically significant finding of this study lies in the notable heterogeneity in safety profiles. MZR demonstrated a distinct dual character. SUCRA rankings clearly indicated that MZR ranked first in terms of the lowest incidence of gastrointestinal AEs, significantly outperforming MMF and MPA. This is a critically important advantage, as gastrointestinal toxicity is the most common reason for dose reduction or discontinuation of MMF/MPA, directly impacting patient adherence and quality of life. MZR provides an excellent alternative for patients who struggle with treatment due to intolerance to MMF/MPA-related gastrointestinal side effects, thereby facilitating long-term and stable immunosuppressive therapy. Dong et al. (28) reported that oral MZR demonstrated comparable efficacy and safety to CTX in patients with lupus nephritis. Miura et al. (29) observed that IL-6 expression remained unaffected at low concentrations of MZR, while high concentrations suppressed IL-6 expression. In contrast to its gastrointestinal safety advantage, MZR ranked lowest (16.2%) in the SUCRA ranking for BK viremia, suggesting it may carry the highest risk in this regard. This aligns with MZR's pharmacological mechanism of action. As a purine analog, MZR may exert differential effects on lymphocyte subsets, including effector cells involved in viral control, relative to MMF, potentially altering the risk profile for viral infections. This finding serves as a clinical warning: for patients treated with MZR, proactive monitoring of BK viremia (e.g., regular plasma BK virus DNA testing) is essential to enable early detection and intervention, thereby preventing BK virus-associated nephropathy (BKVAN) and subsequent graft injury. Negligible difference existed in leukopenia incidence between MZR and MMF, and MZR demonstrated the highest SUCRA ranking, indicating a controllable risk of hematological toxicity, potentially even superior to other agents. This is a reassuring finding. MZR is not merely a substitute for MMF/MPA but rather a differentiated therapeutic option with a distinct safety profile. Clinicians should perform individualized benefit-risk assessments when selecting immunosuppressants. For patients with poor gastrointestinal tolerance or compromised adherence, MZR represents a highly attractive alternative, capable of improving quality of life while maintaining immunosuppressive efficacy. Conversely, for patients receiving MZR, a more rigorous and proactive BK virus monitoring strategy must be implemented to mitigate its potentially higher risk of viral infection. Matsuoka et al. (30) reported that, in patients with systemic lupus erythematosus, MMF did not drastically enhance risk of severe infections vs. other immunosuppressants. MMF may also facilitate reduction of prednisolone dosage and has established itself as a valuable immunosuppressive agent (31).

This study provides important insights; however, several limitations should be acknowledged. The study number and sample size were limited, particularly for analyses of certain outcomes (such as BK viremia), which may have reduced statistical power and affected the stability of the results. Variations in MZR dosing regimens, types and concentrations of CNIs, and follow-up durations across the original studies may have introduced heterogeneity. The inability to access individual patient data precluded more in-depth dose-response or subgroup analyses. In this work, the Cochrane RoB 2.0 tool was employed for methodological quality assessment. For intuitive presentation in the result figures, conventional terminology (e.g., selection bias, detection bias) was retained; however, the assessment itself remained grounded in the RoB 2.0 framework, which does not affect the overall quality evaluation conclusions. Future research should focus on conducting well-designed, large-scale head-to-head RCTs to directly compare the long-term outcomes and specific safety events (especially BK viremia) of MZR and MPA. Additionally, studies exploring the optimal therapeutic window for MZR concentration monitoring, its synergistic effects with different CNI combinations, and the identification of risk factors for BK viremia will hold significant clinical value.

## 5 Conclusion

This NMA demonstrates that, within CNI-based maintenance regimens, MZR is non-inferior to MPA and MMF in terms of both patient survival and graft survival over a 3-year follow-up period. Although the 3-year survival rate is often regarded as an important indicator of mid- to long-term efficacy in transplantation research, longer-term follow-up data are still required to confirm its true long-term therapeutic benefits. However, MZR presents a distinct safety profile: its gastrointestinal tolerability is significantly superior to that of MMF/MPA, making it an ideal alternative for patients intolerant to MMF/MPA due to gastrointestinal adverse effects. Conversely, it may be related to higher risk of BK viremia, necessitating enhanced proactive monitoring for such infections in clinical practice. Therefore, Therefore, MZR should not be regarded merely as a substitute for MMF/MPA, but rather as a distinct therapeutic option with a differentiated safety profile. Clinical decision-making should involve individualized benefit-risk assessment: MZR represents a highly valuable option for patients prioritizing improved quality of life and gastrointestinal tolerability, while those treated with MZR require stringent BK virus monitoring and management strategies.

## Data Availability

The original contributions presented in the study are included in the article/supplementary material, further inquiries can be directed to the corresponding author.
